# Inflammation-related prognostic markers in resected hepatocellular carcinoma

**DOI:** 10.3389/fonc.2023.1267870

**Published:** 2023-12-07

**Authors:** Fabio Giannone, Nevena Slovic, Patrick Pessaux, Catherine Schuster, Thomas F. Baumert, Joachim Lupberger

**Affiliations:** ^1^Université de Strasbourg, Inserm, Institut de Recherche sur les Maladies Virales et Hépatiques Unité Mixte de Recherche (UMR)_S1110, Strasbourg, France; ^2^Unité de Chirurgie Hépato-Biliaire et Pancréatique, Service de Chirurgie Viscérale and Digestive, Hôpitaux Universitaires de Strasbourg, Strasbourg, France; ^3^Institut Hospitalo-Universitaire (IHU), Strasbourg, France; ^4^Service d’hépato-gastroentérologie, Hôpitaux Universitaires de Strasbourg, Strasbourg, France; ^5^Institut Universitaire de France (IUF), Paris, France

**Keywords:** HCC, biomarkers, genetic signatures, inflammation, patient outcome

## Abstract

Hepatocellular carcinoma is usually detected late and therapeutic options are unsatisfactory. Despite marked progress in patient care, HCC remains among the deadliest cancers world-wide. While surgical resection remains a key option for early-stage HCC, the 5-year survival rates after surgical resection are limited. One reason for limited outcomes is the lack of reliable prognostic biomarkers to predict HCC recurrence. HCC prognosis has been shown to correlate with different systemic and pathological markers which are associated with patient survival and HCC recurrence. Liver inflammatory processes offer a large variety of systemic and pathological markers which may be exploited to improve the reliability of prognosis and decision making of liver surgeons and hepatologists. The following review aims to dissect the potential tools, targets and prognostic meaning of inflammatory markers in patients with resectable HCC. We analyze changes in circulant cellular populations and assess inflammatory biomarkers as a surrogate of impaired outcomes and provide an overview on predictive gene expression signatures including inflammatory transcriptional patterns, which are representative of poor survival in these patients.

## 1 Introduction

Hepatocellular Carcinoma (HCC) is the most common primary liver cancer accounting for about 80% of all cases and it ranks as the third leading cause of cancer deaths worldwide (1). Like cholangiocarcinoma, HCC shows a dismal prognosis with a relative 5-year survival rate of approximately 20% (2). Despite the constant and progressive evolution of the therapeutic algorithms on which decision strategy is based, in clinical practice several issues remain to be addressed. First, a reliable prognostic clinical marker to predict HCC outcome is still missing. Among the prognostic indicators, the most common is plasmatic alpha-protein (AFP), which correlates with tumor behavior and risk of recurrence and survival (3–5). However, in 15–30% of HCC, AFP levels remain in a normal range and the heterogeneity of studies prevents from formulating clear recommendations (6, 7). Secondly, the complex treatment allocation process does not always reflect in a complete therapeutic arsenal. Effective and validated peri-operative therapies are still lacking and the inability to accurately detect more aggressive tumors could lead surgeons to validate complex and high morbidity resections on patients with an elevated risk of recurrence (8). In the last years several authors reported a strong correlation between systemic inflammation and HCC prognosis with different systemic and pathological markers associated with survival and recurrence. For example, high values of platelet-to-lymphocyte ratio (PLR), neutrophil-to-lymphocyte ratio (NLR) and other similar scores seem to predict poor long-term outcomes after treatment (9–11). This relationship is also evident on a molecular level as gene expression alterations are at the basis of these inflammatory cell shifts on which cancer develops and progresses (12). In this review, we provide a comprehensive overview and update on the prognostic meaning of inflammatory modifications in patients with resectable HCC. We analyze changes in circulant cellular populations and assess inflammatory biomarkers as a surrogate of impaired outcomes and provide an overview on predictive gene expression signatures including inflammatory transcriptional patterns, which are representative of poor survival in these patients.

## 2 Inflammatory microenvironment in HCC carcinogenesis and prognosis

**Figure 1:**
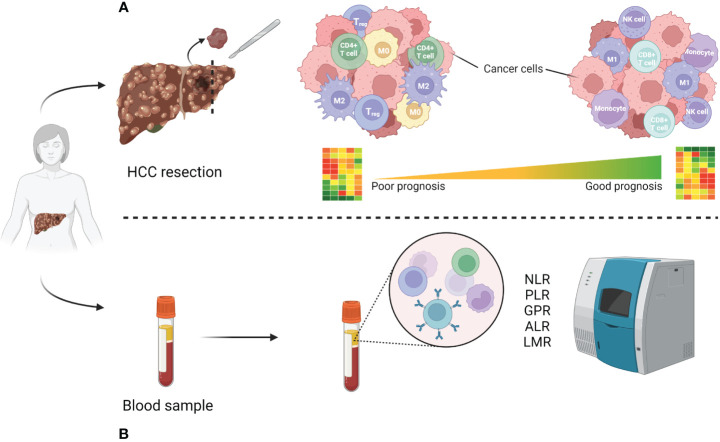
*Immune cell population difference analysis in poor vs good prognosis patients*. **(A)** High-risk resected patient tissue with poor prognosis tends to be enriched with regulatory immune cells (Treg, CD4+ T cell), type 2 macrophages (M2) as well as non-activated macrophages (M0), as opposed to natural killer (NK) cells, CD8+ cytotoxic T cells, type 1 macrophages (M1) and monocytes in good prognosis patients (29–31). **(B)** Most used inflammatory markers analyzable from patient blood samples. Created using BioRender.

A large body of knowledge has demonstrated that a dysregulation in tumor microenvironment (TME) contributes to carcinogenesis and tumor progression (13). Chronic inflammation is considered as an excessive, abnormal, and prolonged form of cellular immune responses interacting with other factors in the development of the neoplastic process (14). A large panel of innate immune cells in the tumor microenvironment (macrophages, neutrophils, dendritic cells, innate lymphoid cells, myeloid-derived suppressor cells, and natural killer cells) as well as adaptive immune cells (T cells and B cells) are linked to tumor progression and outcome (15). Tumors control their microenvironment by a large number of tumor-associated factors promoting its establishment, growth, survival, and spread by shaping a pro-tumoral local cytokine milieu (15). This cause-effect relationship is well described in HCC patients and several mechanisms have been shown to be related to tumor development, progression, and overall survival. The majority of HCCs occur in injured liver after stimulation with different inflammation-triggering agents, as viruses, alcohol, drugs, toxins, or obesity (16). Alterations in inflammatory cell populations and a dysregulation of genes and protein expression pattern have been correlated with long-term outcomes in HCC patients. Among many others, these involve an upregulation of several metalloproteinases (MMP) and downregulation of C-type Lectin-like Receptor 2 (CLEC2) which were found to be associated with impaired survival (17). Similarly, hyperexpression of PD-1 and PD-L1 in neoplastic hepatocytes and lymphocytes infiltrating the tumor is a marker of poor survival, while in slowly growing HCC these markers are barely expressed (17). Other authors demonstrated that TNF, IL6 and CCL2 mutations are those most significantly associated with outcomes and considerably longer survival was seen in patients with higher levels of both TNF and IL6 (18, 19). To our knowledge, out of the mentioned markers, targeted therapies have been developed for PD-1 and PD-L1, while the clinical trials targeting the other mentioned markers have so far been unsuccessful, at least in the context of HCC (20–27). The above-mentioned markers have been summarized in **Table 1**. In regard to cell populations (**Figure 1**), Kuang and co-workers found that peritumoral stroma of HCC tissues was enriched with neutrophils and their levels could serve as a powerful predictor for poor survival in HCC patients (32). Accordingly, high inflammatory cytokine levels in the tumor can promote local and systemic neutrophilia (33). Lymphocytes are at the same time involved in tumor progression, and an enhanced infiltration of specific subtypes within the tumor samples, as CD8+ and CD3+ T cells, CD20+ B cells and CD56+ NK cells, was found to be present in patients with longer survival (18, 34). A recent study (28) identified a structure formed by specific cell populations and its role in immunotherapy resistance. It was found that a subpopulation of macrophages with high expression of osteopontin (SPP1), in combination with CAFs (cancer-associated fibroblasts) mediates resistance to immune checkpoint inhibitors. Blocking SPP1, a phosphoprotein with a previously identified regulatory role in the TME (35), rendered the tumors more responsive to immunotherapy in an animal model. It was therefore marked as a target for further clinical studies in the context of HCC, but to our knowledge, no such trials are currently in progress. It is also worth noting that this study focused on a restricted number of cases and did not explore the potential of SPP1 as a serum inflammatory marker.

**Table 1:** Markers of the inflammatory microenvironment of HCC patients.

Type of marker	Study	Expression change	Prognostic meaning
MMP1, MMP10, MMP12	Critelli et al., 2017 (17);	Upregulation	Decreased Survival
CLEC2	Critelli et al., 2017 (17);	Downregulation	Decreased Survival
PD1	Critelli et al., 2017 (17);	Upregulation	Decreased Survival
PDL1	Critelli et al., 2017 (17);	Upregulation	Decreased Survival
TNF	Chew et al., 2010 (19); Chew et al., 2012 (18)	Upregulation	Increased Survival
IL6	Chew et al., 2010 (19); Chew et al., 2012 (18)	Upregulation	Increased Survival
CCL2	Chew et al., 2010 (19); Chew et al., 2012 (18)	Upregulation	Increased Survival
SPP1	Liu et al., 2023 (28)	Upregulation	Decreased Survival

## 3 Serum inflammatory markers

Based on the strong association between tumor microenvironment and natural history of tumors, modifications in circulating inflammatory markers highlight more aggressive diseases and therefore predict poor outcomes. These patterns have been implemented in clinical practice as scores, which have the advantage of being easy to approach, calculated with routine laboratory tests, thus with limited costs, and available before surgical treatment. The most diffused and described serum inflammatory marker in resected HCC is undoubtedly the NLR (10, 11, 36–40). An increased NLR, despite the different cut-offs used by the authors, seems associated with reduced overall survival and disease-free survival rates after curative resection. Neutrophil count, rather than reduced lymphocytes, could probably explain these results, knowing that elevated neutrophils associated independently with poorer survival and impaired performance status in HCC (41). Although other publications did not support the prognostic value of NLR at univariate or multivariate analysis (11, 36, 40, 42), two meta-analyses confirmed the significant correlation with impaired prognosis in resected patients (43, 44). Another well-established immunity-related score found to be predictive of long-term outcomes in resected HCC is the PLR. Several studies confirmed a strong association between oncologic outcomes and an elevation of this index and, unlike NLR, this biomarker has almost always confirmed its prognostic role at multivariate analysis (10, 11, 38–40, 44, 45). Other less explored scores are the gamma-glutamyl transpeptidase-to-lymphocyte ratio (GLR) (11, 36), the aspartate aminotransferase-to-lymphocyte ratio (ALR) (11, 46) or the lymphocyte-to-monocyte ratio (39, 40), all more or less related to long-term outcomes. A summary of these inflammatory biomarkers as well as studies assessing their prognostic role is shown in **Table 2**. In order to increase the accuracy of these biomarkers, some authors developed new scores by combining these aforementioned values together or by adding other non-inflammatory variables in the formula. The first group includes indexes as the A-G-P score, a predictive model to accurately predict survival by analyzing at the same time the ALR, the GLR and the PLR (11). This equation demonstrated to be an excellent independent predictor of OS in resected patients and, at the same time, being able to stratify patients with HCC according to the resulting score well (11). On the other hand, other formulas have been developed starting from these inflammatory markers and other serum values, as nutritional indexes. This is the case of the Glasgow prognostic score (GPS) and the modified GPS, calculated from the CRP and the albumin level (47, 48), the prognostic nutritional index (PNI) combining lymphocyte count and serum albumin (29, 49) or the inflammation-immunity-nutrition score (IINS), a combination of CRP, lymphocyte count and serum albumin level (30). All these equations, although not systematically integrated in clinical practice, have been widely described as factors of impaired survival in literature.

**Table 2:** Prognostic meaning of different serum inflammatory markers in resected hepatocellular carcinoma in aforementioned studies.

Type of marker	Study	Cut-off assessed	Number of patients	Prognostic meaning	Role at multivariate analysis
Neutrophil to lymphocyte ratio (NLR)	Sullivan et al., 2014 (42)	–	75	Not predictive of OS	–
	Lu et al., 2016 (37)	2.81	963	Shorter OS and RFS	Independent risk factor for OS and RFS
	Zheng et al., 2017 (39)	–	370	Shorter OS and RFS	Lost
	Wang et al., 2019 (10)	2.92	239	Shorter OS and RFS	Independent risk factor for OS and RFS
	Dai et al., 2020 (36)	2.5	302	Shorter OS and DFS	Lost
	Wu et al., 2021 (11)	2.33	347	Shorter OS, no differences in DFS	Lost
	Silva et al., 2022 (38)	1.715 for OS 2.475 for DFS	161	Shorter OS and DFS	Lost
	Zhou et al., 2022 (40)	4.191 for OS 2.271 for RFS	91	Shorter OS, no differences in RFS	Lost
Platelets to lymphocyte ratio (PLR)	Zheng et al., 2017 (39)	275 for RFS a 298 for OS	370	Shorter OS and RFS	Independent risk factor for OS and RFS
	Wang et al., 2019 (10)	128.1	239	Shorter OS and RFS	Independent risk factor for OS and RFS
	Wu et al., 2021 (11)	117.09	347	Shorter OS, no differences in DFS	Independent risk factor for OS
	Kim et al., 2022 (45)	132	159	Shorter OS and RFS	Independent risk factor for OS
	Silva et al., 2022 (38)	115.05 for OS 100.25 for DFS	161	Shorter DFS	Independent risk factor for DFS
	Zhou et al., 2022 (40)	302.104 for OS 228.644 for RFS	91	Shorter OS and RFS	Independent risk factor for OS and RFS
Gamma-glutamyl transpeptidase to platelet ratio (GPR)	Dai et al., 2020 (36)	0.35	302	Shorter OS and DFS	Independent risk factor for OS and DFS
	Wu et al., 2021 (11)	0.48	347	Shorter OS and DFS	Independent risk factor for OS and DFS
Aspartate aminotransferase to lymphocyte ratio (ALR)	Chen et al., 2021 (46)	26.6 for OS 27.9 for RFS	983	Shorter OS and RFS	Independent risk factor for OS and RFS
	Wu et al., 2021 (11)	31	347	Shorter OS and DFS	Independent risk factor for OS and DFS
Lymphocyte-to-monocyte ratio (LMR)	Zheng et al., 2017 (39)	–	370	Shorter OS and RFS	Lost
	Zhou et al., 2022 (40)	3.785 for OS 4.633 for RFS	91	No differences	–

OS, Overall Survival; RFS, Recurrence-Free Survival; DFS, Disease-Free Survival.

## 4 Gene signatures

An emerging toolset potentially complementing the classical predictive markers in the clinics are transcriptional gene signatures (GS). They refer to expression values of a group of genes, and are mostly representative of a condition, healthy, diseased or both. The expression pattern of genes is often correlated with the activity of their products and can therefore infer on the cell processes these genes are a part of. Recent technological advancements enable the collection and analysis of large quantities of biological data, as in cases of gene expression values across the genomes of multiple cells. This kicked off the development of gene signatures in several diseases and cancer. Majority of GS have been assessed as predictive tools and are derived from data obtained using techniques such as quantitative PCR (qRT-PCR), hybridization arrays (oligonucleotide, cDNA), RNA sequencing etc., that all have the analysis of levels of RNA production in common. Most signatures focus on messenger RNA transcription, while some of them are based on microRNA (miRNA) (31), long non-coding RNA (lncRNA) (50) or protein expression (51).

Contrary to most classical prognostic pathological or clinical features, the analysis of gene signatures allows a profound molecular profiling of the tumour environment. As cancer is a multicellular disease often involving several systems within the body, analysing gene expression patterns from multiple cell types facilitates identification of dysregulated pathways and their comprehension. Gene signatures provide a list of differentially expressed genes (DEG), upregulated or downregulated between the compared groups, usually diseased and non-diseased or healthy conditions. Tissues that are presumably not affected but surrounding the cancer area are usually considered as non-diseased, while healthy tissue is obtained from regions distant from the affected area. Out of the selected genes, some are linked to a poor prognosis or high risk while others are marked as good-prognosis or low risk genes. Therefore, the combination of both poor and good prognosis gene expression pattern allows a classification of patients into high and low-risk groups. The predictive capacity of a signature is mostly measured using machine learning-derived ROC (Receiver Operating Characteristics) and AUC (Area Under the ROC Curve) values, while some authors also use confidence intervals. The closer the AUC value is to one, the more accurate the predictive signature is (52). Recent analyses have studied the drawbacks of gene signatures, notably their redundancy and possibilities of improving them (53). Even with drawbacks, these signatures can be efficient for a statistically important number of patients and therefore their use in clinical practice should not be ignored.

### 4.1 Gene signatures predicting HCC recurrence and survival in resected patients

To date, most signature-based studies focus on predicting recurrence as well as survival in HCC patients. A study from 2020, found that 66% of patients experienced HCC recurrence over a period of 8 years emphasizing the drastic recurrence rates of HCC (54). Although still debated, the classification of tumor recurrence into early and late recurrence is strongly linked to the tumour origin. Secondary tumours originating from leftover cancer cells of the resected tumour within two years after surgery are defined as early recurrence, whereas tumours originating from novel cancer cells of the same organ (*de novo* tumour) more than two years after surgery are considered as events of late recurrence (55).

As early as 2008, the first collection of 186 genes was published in the pioneering work from Hoshida et al., highlighting 73 poor and 113 good prognosis genes being predictive for survival in liver disease (56–59). The authors established a robust signature of DEGs from tissues surrounding HCC of 106 resected patients which was then validated in another cohort of 234 patients. They managed to overcome the technical difficulty to analyse more commonly available formalin- and paraffin-treated (FFPE) tissues instead of depending on snap frozen tissues. This signature has since been further studied and validated in additional cohorts. A 5-gene signature from frozen liver tissues was reported (TAF9, RAMP3, HN1, KRT19, and RAN) predicting survival from HCC in 314 HCC patients (60). Depending on the differential expression of these five genes, patients were stratified into poor and good prognosis groups, and the signature was validated in external cohorts of patients. As reported by Nault and co-workers (60), the comparison of the two signatures described above validated the findings from both articles, also as the signatures provide similar output, i.e., a comparable stratification of patients in their corresponding poor and good survival groups. More recently, a signature specific for early recurrence in HCC has been described, which was not based on coding genes but on 25 lncRNAs, another type of RNA relevant in HCC development (50). This signature had better predictive performance than multiple other factors, including serum AFP. Interestingly, the high and low-risk groups correlated with the immune characterization of the tissue of these patients; for example, the low-risk group showed higher levels of tumour-infiltrating lymphocytes. Another 9-gene survival signature with links to immune microenvironment was derived from the analysis of 274 resected HCC patient tissues by another group (61). Of the four upregulated (C2HC1A, MARCKSL1, PTGS1, CDKN2B) and five (CLEC10A, PRDX3, PRKCH, MPEG1, LMO2) downregulated genes in poor prognosis patients, several signature genes have direct or indirect roles in cancer immune environment (CLECL10A, PTGS1, C2HC1A). Even though they focused on data from HCC patients of viral aetiology, their established signature is seemingly outperforming the previously established ones (61). Finally, a more recurrence-specific gene signature had been identified by comparing recurrence and non-recurrence HCC tissues from 85 patients (62). Within the selected genes, two (HMGA1 and RACGAP1) were found to be particularly relevant for recurrence in HCC patients. Interestingly, both genes have recently been studied for their role in cancer immunity (63, 64). However, while some of the signature genes are known to have roles in HCC, they are generally parts of unrelated pathways and do not necessarily interact with each other.

### 4.2 Inflammatory gene signatures

As single-cell resolution in transcriptomic analysis boosted our understanding of the HCC microenvironment (65, 66), signatures derived from immune cell populations or linked to immunity in HCC have been increasingly explored in the recent years. However, most of these studies tend to use a variety of patient tissues as source, including results from not only resected patients, but also biopsies of advanced HCC or data found in online databases, mainly from The Cancer Genome Atlas (https://www.cancer.gov/tcga). To our knowledge, resection-specific immune gene signatures have yet to be established. A study from 2021 established a robust immune-related gene signature containing seven genes from TCGA-derived data of 372 patients with a variety of backgrounds (histological grade, clinical stage, survival rate etc.) (67). Six out of seven genes (S100A8, BIRC5, CACYBP, NR0B1, RAET1E, SPP1) were associated with high-risk of survival, while SPINK5 was identified as a low-risk factor. On the cellular level they found that immunosuppressive cell groups such as CD4+, Treg cells, M0 and M2 macrophages, as well as neutrophiles were more abundant in the high-risk groups compared to the low-risk ones (**Figure 1**). This signature, however, needs further testing before it can be confidently applied in patients. Another recent study used a similar but more focused approach, as they report developing an eight gene signature based on M2-like tumour associated macrophages from both patient biopsies and resections (68). Similar findings were reported by two independent studies, whose 6 and 8 immune-related gene signatures had an AUC of 0.71 and 0.68, respectively (69, 70). Finally, Shi and co-workers reported a non-invasive immune signature for early-stage HCC based on the analysis of cells from patient blood samples using single cell cytometry (65). In this dynamic immune atlas, they identify mainly lymphocyte (sub)types characterizing advanced stages of HCC using only patient blood samples. In general, most recent immune signatures tend to have less than 10 genes and their AUC values vary from 0.65 to 0.75. These have been summarized in **Table 3**. Of note, all the listed studies report the tendency of presence of contrasting immune cell types within the high-risk compared to low-risk group: the high-risk patient group tends to be enriched with macrophages and Tregs while B, NK, cytotoxic T cells and mast cells are less represented.

**Table 3:** A summary of immune-related predictive signatures in HCC: their predictive power, data origin and the defined genes.

Signature	Study	AUC	Good/Poor prognosis genes	Data origin	Patients
An Inflammatory Response-Related Gene Signature Can Impact the Immune Status and Predict the Prognosis of HCC	Zhuo et al., 2021 (69)	0.685, 0.626, 0.605 at 1, 2, and at 3 years	SERPINE1	TCGA LIHC&ICGC	>400
			ADORA2B, MEP1A, P2RX4, ITGA5, NOD2, RIPK2, SLC7A		
Survival prediction and response to immune checkpoint inhibitors: A prognostic immune signature for HCC	Ying et al., 2021 (70)	0.71 at 5-year survival	FYN, IGF1, MASP1, NR3C2, TGFBR3	TCGA&GEO	>400
			BIRC5		
Identification of a prognostic and therapeutic immune signature associated with HCC	Peng et al., 2021 (67)	0.77, 0.73, and 0.74 in predicting 1-, 3-, 5-year overall	SPINK5	TCGA, GEO & ICGC	>400
			BIRC5, CACYBP, NR0B1, RAET1E, S100A8, SPP1		
M2-like tumor-associated macrophage-related biomarkers to construct a novel prognostic signature, reveal the immune landscape, and screen drugs in HCC	Qu et al., 2022 (68)	1, 3, and 5 years was 0.728, 0.689, and 0.663,	KLF2	TCGA, GEO & ICGC	>400
			LIM3, PAM, PDLIM7, FSCN1, DPYSL2, ARID5B, LGALS3		
Single-cell immune signature for detecting early-stage HCC and early assessing anti-PD-1 immunotherapy efficacy	Shi et al., 2022 (65)	–	Cells, no genes specified	PBMC at resection	~50

TCGA, The Cancer Genome Atlas; LIHC, Liver Hepatocellular Carcinoma; ICGC, International Cancer Genome Consortium; GEO, Gene Expression Omnibus; PBMC, peripheral blood mononuclear cells.

## 5 Challenges & future directions

Inflammation is a key player in the natural history of HCC and thus the relationship between some inflammatory-based tools and patients’ prognosis are closely linked by the disease biology of hepatocarcinogensis (71–74). This observation offers an opportunity to predict long-term outcomes as precise as possible if compared to current markers. Although some of the biological markers above cited clearly show a direct and independent connection with recurrence and survival after liver resection for HCC, they are far from being extensively implemented in clinical practice. Limitations of the currently available serum biomarkers are the difficulty in standardizing reliable cut-offs as well as the universal validation of their prognostic role, regardless of underlying patient pathologies or cancer aetiology. When assessing the above-mentioned ratio (NLR, PLR, etc.), cut-off values are determined by the AUC and therefore always different among all the studies. As a result, we found that authors use various values to define cases with impaired outcomes and, sometimes, these values are significantly different if considering the type of outcome assessed, as recurrence or survival (38, 40). A recent meta-analysis assessing the role of NLR, found that among 13 included studies the cut-off values ranged between 1.505 and 5.0, and only a few studies used the same ratio (43).

Another issue to solve is the large-scale applicability of these markers in clinical practise. This review focusses on resected patients which represent a large minority of all diagnosed HCC. This type of lesion often develops on an immunity-altered host which can distort the results and thus the direct correlation between serum markers and prognosis. Furthermore, authors usually analyze specific subgroups of HCC patients in order to create a homogeneous cohort, as tumors in well-compensated cirrhosis (40). In 2016, Lu et al. assessed the utility of the NLR and used subgroup analysis to examine this potential relationship separately in patients in BCLC stages 0/A, B, or C (37). The authors found that this marker may be a good predictor of survival in early/intermediate stage, whereas it was not associated with risk of overall survival (OS) or tumor recurrence in patients with stage C disease. Similar limitations are found when comparing the potential of transcriptional signatures. Despite the very promising results from a decade of development, no predictive transcriptomic signature is used in a clinical setting. Like the serum biomarkers, the AUC values used to quantify the power of GS vary significantly, and do not have confirmed utility until the signatures are confirmed by other teams or in clinical settings. Also, as we mentioned earlier and for the purpose of this review, resection-specific immune/inflammatory gene signatures have been scarce. However, a promising immune signature has been recently identified using artificial intelligence on transcriptomics of resected patients (75). The authors argue their approach would allow for the bypass of technical bias and restriction induced with a more “classical” gene signature approach. Moreover, patient samples used are often restricted to small numbers, a single country, patient population or aetiology, potentially affecting the applicability of these signatures without validation in other cohorts (61, 62, 68). An additional important limitation of the transcriptional signatures is their dependency on patient liver tissues. Non-invasive methods, such as described by Shi and co-workers (65), should thus be prioritized in the future. Initiatives to translate transcriptional signatures into minimal-invasive blood surrogates have already been taken with a recently published eight-protein signature termed PLSec (76). It is based on the 186-gene PLS (56, 58) and is predictive for survival, as well as recurrence of HCC in advance fibrosis patients. The very encouraging data are based on the analysis of 400 patients in total and pave the wave for further consolidation in larger cohorts. Out of the eight, 6 proteins were marked as high-risk, including vascular cell adhesion molecule 1 (VCAM-1), insulin-like growth factor-binding protein 7 (IGFBP-7), gp130, matrilysin, IL-6, and C-C motif chemokine ligand 21 (CCL-21), and 2 were defined as low-risk-associated serum proteins, angiogenin and protein S. Collectively, new combinations of classical and novel blood-based biomarker signatures will likely have the biggest impact in transforming patient care.

Finally, beyond the pure prognostic meaning, another non-negligible potential of these biomarkers lies undoubtedly in the possibility of guiding therapeutic approaches in advanced disease. Finding a biomarker which could accurately predict tumor progression or response to specific treatments would mean opening the door to precision medicine in HCC, as already established in other cancers (77). Although immunotherapy is the first-line option in these patients, with drugs targeting different checkpoints of the immune system, no correlation between tissue and serum inflammatory markers and chemotherapy sensibility have been demonstrated in literature to date. Other non-inflammatory biomarkers have been tested with usually poor or not significant results (78). Currently, there is no established role or indication for molecular or genetic testing in HCC due to the absence of specific benefit. Only a few mutations can influence the therapeutic algorithm in HCC but exclusively in case of progression after first-line administration, and in certain circumstances (79). Similarly, ramucirumab, another second-line option, has shown better outcomes in advanced HCC with AFP > 400 ng/ml previously treated with sorafenib, leading international drug agencies to approve this anti-VEGF drug in this setting (80). However, the restriction of ramucirumab to patients with AFP > 400 ng/ml does not mean that this should be the agent of choice for that population (81). Further trials are therefore urgently needed to identify new biomarkers for precision medicine in HCC.

## Author contributions

FG: Conceptualization, Investigation, Writing – original draft, Writing – review & editing. NS: Conceptualization, Investigation, Writing – original draft, Writing – review & editing. PP: Validation, Writing – review & editing. CS: Supervision, Writing – review & editing. TB: Conceptualization, Funding acquisition, Methodology, Supervision, Validation, Visualization, Writing – review & editing. JL: Conceptualization, Methodology, Supervision, Validation, Visualization, Writing – review & editing.
